# Antiphospholipase A2 Receptor Autoantibodies: A Step Forward in the Management of Primary Membranous Nephropathy

**DOI:** 10.1155/2015/249740

**Published:** 2015-10-20

**Authors:** Bogdan Obrisca, Gener Ismail, Roxana Jurubita, Catalin Baston, Andreea Andronesi, Gabriel Mircescu

**Affiliations:** ^1^“Carol Davila” University of Medicine and Pharmacy, Dionisie Lupu Street No. 37, District 1, 022328 Bucharest, Romania; ^2^Center of Internal Medicine-Nephrology, Fundeni Clinical Institute, 258 Fundeni Street, District 2, 022328 Bucharest, Romania; ^3^Center for Uronephrology and Renal Transplantation, Fundeni Clinical Institute, 258 Fundeni Street, District 2, 022328 Bucharest, Romania; ^4^“Dr. Carol Davila” Teaching Hospital of Nephrology, Calea Grivitei Street No. 4, District 1, 010731 Bucharest, Romania

## Abstract

Since the identification of PLA2R (M-type phospholipase A2 receptor) as the first human antigenic target in primary membranous nephropathy (MN), perpetual progress has been made in understanding the pathogenesis of this disease. Accumulating clinical data support a pathogenic role for the anti-PLA2R antibodies (PLA2R ABs), but confirmation in an animal model is still lacking. However, PLA2R ABs were related to disease activity and outcome, as well as to response therapy. Accordingly, PLA2R ABs assay seems to be promising tool not only to diagnose MN but also to predict the course of the disease and could open the way to personalize therapy. Nevertheless, validation of a universal assay with high precision and definition of cut-off levels, followed by larger studies with a prolonged follow-up period, are needed to confirm these prospects.

## 1. Introduction

Despite the increasing prevalence of the focal and segmental glomerulosclerosis in certain subpopulations [1–5], primary membranous nephropathy (MN) is still the leading cause of adult nephrotic syndrome in the Caucasian populations [6–8]. Primary membranous nephropathy is a glomerulus-specific autoimmune disorder in which subepithelial* in situ* formation of immune complexes injures the glomerulus [9].

The landmark papers identifying the M-type phospholipase A2 receptor (PLA2R) [10] and the Thrombospondin Type-1 Domain Containing-7A (THSD7A) [11] as human antigenic targets in adult MN in 70–75% and, respectively, 2.5–5% of cases restricted the designation of “idiopathic” disease to a minority of cases.

## 2. PLA2R AB and MN Pathogenesis

PLA2R is a type I transmembrane glycoprotein, member of the mannose receptor (MR) family. Characteristically, all four members of the MR family have a large extracellular glycosylated region comprising an N-terminal cysteine-rich domain (CysR), a fibronectin-like type II domain (FnII), and eight to ten C-type lectin-like domains (CTLD_1–10_) [12–14]. PLA2R serves primarily as a receptor for secretory PLA2, allowing its removal from circulation, thus regulating its biological effect [15–17].

As in many other autoimmune diseases, the triggering event of anti-PLAR2 and anti-THSD7A autoantibodies formation is still a matter of debate. Beck et al. [10] observed that anti-PLA2R antibodies recognize their target antigen only under nonreducing conditions suggesting that PLA2R contains a conformation-dependent epitope. Kao et al. [18] were the first to describe the location of the immunodominant epitope within PLA2R. They observed that a three-domain protein complex—consisting of CysR, FnII, and CTLD1—is recognized by sera from patients with MN. In addition, absence of either CysR or CTLD1 domain rendered the remaining fragments without any antigenicity, thereby supporting the critical importance of these two domains. It appears that CTLD1 is essential for stabilizing the structure of this epitope given the presence of a disulfide bond between CTLD1 and FnII which explains, at least in part, the sensitivity to reducing conditions.

Later on, Fresquet et al. [19] described eight peptides, located in the CysR, FnII, CTLD3, and interdomain loops between CTLD 1/2 and CTLD 2/3, as potential constituents of the PLA2R major epitope. These peptides are discontinuously spread in the primary structure of the protein but are brought in proximity through disulfide bonds in the tertiary structure, forming the three-dimensional configuration characteristic of the epitope. A more careful analysis revealed that only two of these peptides, located in a close region in CysR, possess the ability to successfully bind to anti-PLA2R antibodies, thereby defining the major epitope in PLA2R.

However, it is still unknown what sets up the immunogenicity of this antigen. A complex interplay of genetic and probably environmental factors could be the pathogenic trigger for MN. Genetic variants within the coding region of the PLA2R gene on chromosome 2 strongly associated with the development of MN were identified by genome-wide analyses. However, these single nucleotide polymorphisms are also frequently found in the general population, contrasting with the rarity of this disease [20, 21]. The intervention of environmental factors, not yet identified, could induce structural changes of PLA2R or expression of its hidden epitopes, making it antigenic [22]. The combined intervention of these factors could lead to the expression of PLA2R with a specific amino acid sequence, allowing for a particular three-dimensional conformation capable of activating the innate immune system.

The dendritic cells will intercept the modified epitopes of PLA2R and will then present them in association with the HLA protein to the cells of adaptive immune system [22]. Single nucleotide polymorphisms of HLA-DQA1 genes on chromosome 6 were also associated with MN [23] and it was suggested that the modified antigen presenting protein HLA-DQA1 could be involved in the initiation of an autoimmune response targeting variants of PLA2R1 [22]. In addition, molecular mimicry could play a role, as peptides of PLA2R showed partial homology with bacterial cell wall enzyme common to* Clostridium* species [24, 25].

**Figure 1:**
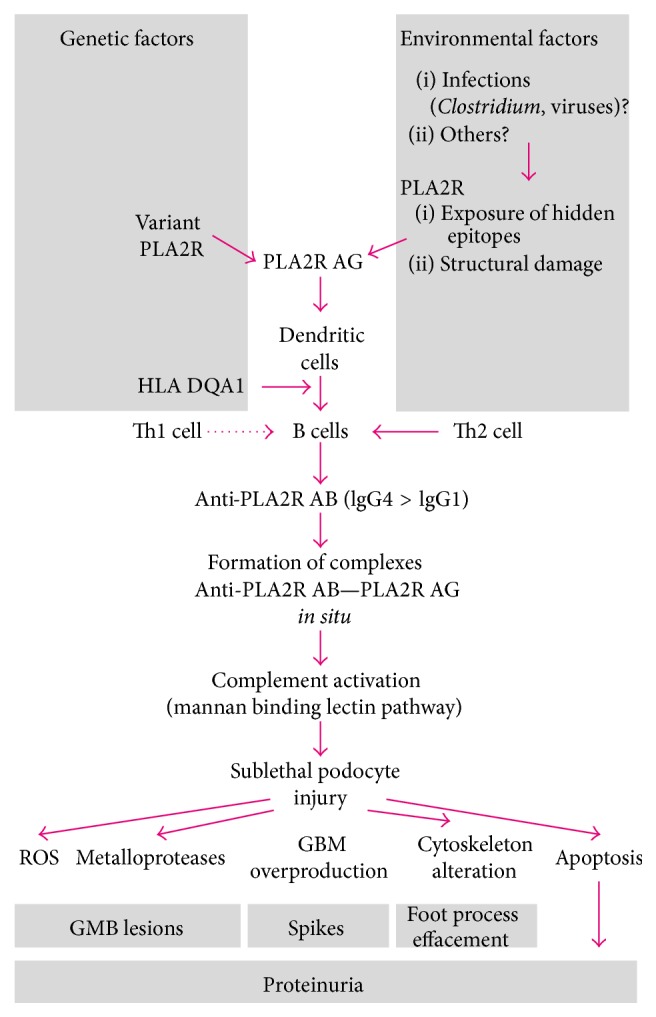
Phospholipase A2 receptor (PLA2R), anti-PLA2R antibody (anti-PLA2R AB), and membranous nephropathy pathogenesis. GBM: glomerular basement membrane; ROS: reactive oxigen species.

The main characteristic of immune response in MN is the predominant activation of Th2 lymphocytes, which in turn will produce certain cytokines, especially IL-4, IL-10, and IL-13 [26–28]. IL-4 will subsequently activate the B-lymphocytes to synthetize IgG4, the main immunoglobulin subclass found in MN [10, 29]. IgG4 will bind to the conformational epitope of PLA2R1 located on the podocyte surface, forming the characteristic subepithelial immune complexes (Figure 1).

Although the current evidence of the role of anti-PLA2R antibodies in the pathogenesis of MN has enlightened our understanding of the disease, a transgenic animal model of MN is still needed for a direct proof of a pathogenic link between PLA2R ABs and MN. This approach could offer some answers regarding which subtype of immunoglobulin or complement pathway is majorly involved in this disease, which is the actual trigger of this autoimmune process, and why the clinical manifestations are limited to the kidney, although PLA2R is also expressed on alveolar type II epithelial cells and on leukocyte surface [10, 18].

The formation of immune complexes and their accumulation in electron-dense deposits disrupt the functional integrity of the glomerular filtration barrier by a complement-dependent process. However, IgG4 does not activate the complement. As mannose-binding lectin (MBL) was identified in the glomeruli of patients with MN [30] but C1q (a marker of activation by the classical pathway) was not, it was hypothesized that the MBL pathway of complement activation is mainly implicated. Mannose-binding lectin is a member of the collectin family of proteins and, in the presence of calcium, has high affinity for certain oligosaccharides such as N-acetyl glucosamine, mannose, and fucose residues [31]. Another suggestion that immune complexes activate the complement system via MBL pathway comes from the observation that IgG4 anti-PLA2R antibodies have galactose-deficient side chains. Because the galactose molecules are fewer, the N-acetyl glucosamine could be exposed in the terminal position and interact and activate MBL. MBL will then activate two serine proteases (MASP 1/2, MBL associated serine protease), which act similarly to C1r and C1s by splitting C4 (into C4a and C4b) and C2 (into C2a and C2b). C4b and C2a bind together to form C3-convertase, as in the classical pathway, ultimately resulting in the formation of the membrane attack complex (MAC) [31].

However, not all cases of MN seem to share the activation of complement by the MBL pathway. Debiec et al. [32] have described a remarkable case of recurrent MN in an allograft, 13 days after renal transplantation. The graft biopsy specimen showed typical granular staining for C3, C5b-C9, C1q, and IgG3*κ* (with *κ* light chains) and not MBL or IgG4, as would have been expected. A retrospective analysis of the biopsy from the native kidney revealed the same pattern. The presence of monoclonal anti-PLA2R antibodies of IgG3*κ* subclass (which has the highest C1q binding ability) in both native and graft biopsy specimens, together with C1q/C3/C5b-9 deposits, suggests that, at least in this particular case, the classical complement pathway is majorly involved, contrasting with most common forms of MN, in which IgG4 and MBL are the main actors.

After its insertion into cell membranes, MAC is taken up by podocytes, transported intracellularly, and then eliminated in the urinary space [33, 34]. The repair of the cell membrane occurs rather rapidly. Thus, unlike in other situations, in MN, MAC is assembled in sublytic quantities and activates the podocyte rather than destroying it, leading to a series of maladaptative reactions [35]. Overproduction of ROS (reactive oxygen species) by upregulation of NADPH-oxidase will eventually contribute to the alteration of glomerular basement membrane (GBM) as will the synthesis of certain metalloproteases, which disrupt the GBM [36]. Overproduction of extracellular matrix components (type IV collagen, laminin, heparin sulfate, and fibronectin) will thicken the basement membrane and will be deposited around the immune complexes as spike-like extensions. Condensation of actin microfilaments alters the podocyte's cytoskeleton with subsequent effacement of foot processes. Moreover, actin will dissociate from nephrin (the key component of the slit diaphragm), altering the functional integrity of the glomerular filtration barrier. Finally, the apoptosis of podocytes will be favored. All these changes alter the glomerular filtration barrier and result in heavy proteinuria, the main clinical manifestation of MN (Figure 1).

## 3. Could PLA2R ABs Assay Allow Avoiding Biopsy?

Several studies over the past years translated anti-PLA2R antibodies from research laboratories to bedside, by evaluating their diagnostic and prognostic and monitoring potential utilities. Once PLA2R was identified as the major target antigen in MN, the corresponding antibody was regarded as a putative serologic biomarker of the disease. Indeed, patients with active MN were anti-PLA2R positive in 52% and 82% of cases [10, 37–47]. Thus, the specificity seems to be high, around 70–80%, but not 100%, and had variations dependent probably on the assay (Western Blot, indirect immunofluorescence, or ELISA) and of the population cases [10, 37–47] (Table 1). More recently, commercial kits for PLA2R ABs ELISA assay in serum, with excellent concordance with indirect immunofluorescence test, became available [38]. However, although the current immunoassays seem able to detect all PLA2R ABs, the recently described immunodominant epitope within PLA2R could offer the possibility to develop more efficient tools for MN diagnosis [18, 19, 24].

Apart from analytical variation, other factors could explain why specificity of PLA2R ABs is not 100%. In fact, only a high PLA2R ABs titer indicates MN with a high probability, as a low titer test does not exclude MN, since 20% to 30% of the patients with MN are negative for both anti-PLA2R and anti-THSD7A ABs, which appear to be mutually exclusive [10, 11], regardless of the assay used. Other antigens, undetected so far, could also play a role in the pathogenesis of MN. Several other antibodies directed against some intracellular podocyte proteins have been described, such as aldose reductase, superoxide dismutase, and *α*-enolase. Murtas et al. [48] noticed high prevalence of these anti-cytoplasmic antibodies in the serum of patients with MN, of which 10% were positive for all antibodies (including anti-PLA2R antibody) and 20% were negative for all. The coexistence of different circulating anti-podocyte antibodies suggests a complex pathogenesis, which involves several target antigens, some of them still undetected. However, the role of these anti-cytoplasmic antibodies, their translocation from the cytoplasm to the membrane surface, the mechanism of interaction with PLA2R, and their clinical significance are questions that need answers for a better understanding of the disease. Finally, in some cases, proteinuria could persist even after the immunological activity was lost [10], as supported by Svobodova et al. [44] study where 22% of MN patients were anti-PLA2R AB positive, while PLA2R AG was found in 59% of the corresponding biopsies.

On the other hand, in several cases, PLA2R ABs had been detected in the serum of some patients with presumably secondary MN, due to lupus, sarcoidosis, hepatitis B infection, and cancer [49–51]. The reported sensitivity varied between 80 and 100% [10, 41, 43]. However, it is more likely that in such cases MN is coincidentally superimposed on other diseases. This assumption is sustained by the high prevalence of certain neoplasia (lung, colorectal, or gastric) occurring at the age of 40 to 50 years (similar to the onset age of MN) and by the high prevalence of HBV infection in certain geographic regions [37].

Therefore, also an increased PLA2R ABs titer could indicate with a high probability an active MN; the specificity and sensitivity are not high enough to avoid a kidney biopsy. However, PLA2R ABs assay opened a new perspective: at some point, serology will replace histopathology in the diagnosis of MN.

## 4. Could PLA2R ABs at Presentation Predict the Course of MN?

Predicting the clinical course of a patient with MN at disease onset seems rather impossible because of its variable natural history. Classically, the natural history of MN is described by the “rule of thirds” according to which one-third of cases will remit spontaneously, another third will exhibit variable degrees of persistent proteinuria without deterioration in renal function, and the last third comprises the patients who progress to ESRD (end-stage renal disease) [52]. However, it could take from several months to 5 years to achieve a complete (urinary protein excretion <0.3 g/d) or partial remission (urinary protein excretion <3.5 g/d and more than 50% reduction from peak values, accompanied by stable serum creatinine). In this regard, McQuarrie et al. [53] observed that approximately 75% of patients with MN can expect to achieve at least one partial remission (either spontaneous or treatment-induced) within 5 years from diagnosis, contrasting with the traditionally accepted “rule of thirds.”

On the other hand, as immunosuppressive therapy is efficient but not without adverse effects, it should be reserved only to high-risk patients. Serial measurements of proteinuria and serum creatinine are currently used to identify high-risk patients, to predict outcome, and to guide therapy [54]. Various nomograms based on these parameters were designed [55], but more accurate prognostic markers are clearly needed.

The measurements of anti-PLA2R ABs could mark the beginning of the personalized medical management of MN patients, allowing abandoning the empirical approach used so far. The support for this concept comes from several studies, which have tried to evaluate the relation between antibody titers, the disease activity, and the response to therapy.

Several studies confirmed the relationship between PLA2R ABs and indices of MN activity (proteinuria, serum albumin level) first observed by Beck et al. (Table 1) [10, 38, 39, 42, 43]. Hofstra et al. [39, 56] reported a strong correlation between anti-PLA2R AB titers and disease activity, as defined by proteinuria, not only at presentation but also during the follow-up period. They observed that antibody levels were high during the nephrotic phase of the disease, decreased in case of a spontaneous or treatment-induced remission, and increased again at disease recurrence. In addition, the observations that anti-PLA2R antibody levels positively correlated with other markers of kidney injury, such as urinary *β*2-microglobulin, urinary IgG, and serum creatinine, further support the relationship between anti-PLA2R AB titer and disease activity.

More importantly, these authors observed that the antibody titer could predict the clinical outcome, as those with high antibody levels were less likely to achieve a spontaneous remission (4 versus 38%). In another report, patients with higher PLA2R ABs were less likely to have a remission (spontaneous or therapy-induced) and antibody titer, not proteinuria, was an independent risk factor for not achieving remission [46]. Hence, as patients with high PLA2R ABs titer at presentation could have a more active disease and less chances of spontaneous remission, they could benefit from an earlier started immunosuppressive regimen.

## 5. Could PLA2R ABs Guide the Therapy?

In their landmark article, Beck et al. described a temporal relationship between PLA2R ABs and MN activity: the decrease in antibody titers preceded the decline in proteinuria, both in spontaneous and in therapeutic remissions [10]. They considered the observed relation in line with the observations made in the passive Heymann model of nephritis, where proteinuria persisted after the decline in antibody titer, also supporting a pathogenic role for PLA2R ABs: the immunological phase of MN must end before a clinical response is to be seen.

The delayed clinical response could be due to the time needed to operate several processes, for example, clearance of immune complexes, recovery of the podocytes, and repair of slit diaphragm structural damage, which are essential to restore the functional integrity of the glomerular basement membrane.

**Figure 2:**
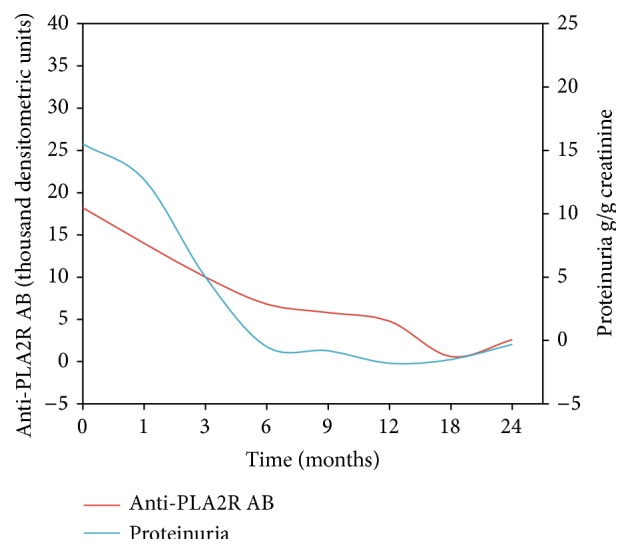
Anti-PLA2R AB and proteinuria in patients with primary membranous nephropathy after rituximab administration (data from Beck et al. [40]).

Two other studies [40, 46] detailed the temporal relationship between the decline in anti-PLA2R antibody titer and proteinuria. In one, the changes in anti-PLA2R ABs levels always preceded the corresponding changes of proteinuria: the decline in antibody titer began rapidly after the start of immunosuppressive therapy, while the corresponding decline in proteinuria occurred slowly and gradually over the following 12–24 months (Figure 2). In the other, the PLA2R ABs titer fell by 81% three months after the initiation of immunosuppressive therapy and remained low thereafter, while proteinuria decreased by only 39% in the first three months but continued to decline slowly in the next 24 months [46]. Therefore, a decline in anti-PLAR2 AB could indicate a forthcoming remission.

Current guidelines recommend monitoring the response to therapy using proteinuria and kidney function (serum creatinine), parameters which describe the consequences of the immunological process. By measuring anti-PLA2R antibody titer, information on the immunological activity of the disease can be obtained and used to monitor the course of MN under therapy.

In this regard, Beck et al. [40] have evaluated the long-term outcome of 35 patients after treatment with rituximab. They observed that 59% and 88% of those with undetectable antibody after 12 months of therapy were in complete or partial remission at 12 and 24 months, respectively, as compared to 0% and 33% among those with persistent antibody. Similarly, Bech et al. [45] observed that antibody levels at the completion of immunosuppressive therapy predicted the long-term outcome: 58% of those with undetectable antibodies at the end of treatment were in persistent remission after 5 years, as compared to none of those with persistent antibodies. Thus, PLA2R-ABs measurements seem to be useful in evaluating the response to therapy and to predict long-term outcome in MN patients.

## 6. How Can PLA2R AB Be Used to Personalize the Management of Primary Membranous Nephropathy Therapy?

First, a low PLA2R ABs titer suggests a less immunologically active disease and could support the decision to delay the initiation of the immunosuppressive therapy [57–60]. Second, in patients with high PLA2R ABs titer, the immunosuppressive therapy seems to be indicated even if proteinuria is lower than 8-9 g/day and before decline in kidney function. Third, the persistence of high antibody titer after several months of therapy should indicate that a switch to a different treatment protocol might be more appropriate. Finally, patients with proteinuria unresponsive to treatment but persistent undetectable serum antibodies are most likely to have an immunologically inactive disease. In these cases, focal segmental glomerulosclerosis secondary to the prolonged disease and tubule-interstitial damage could explain the residual proteinuria and make the immunosuppressive therapy unnecessary. Although these assertions are appealing, one should bear in mind that cut-off values for PLA2R ABs titers are still to be defined and controlled studies should validate these approaches [61].

In conclusion, measurement of anti-PLA2R antibodies is a useful tool for primary MN diagnosis and—in conjunction with other antibodies, still to be defined—would eventually eliminate the need of a kidney biopsy. Anti-PLA2R titers also could guide the therapy as they are related both to immunologic activity and to outcome. Obviously, implementing anti-PLA2R measurements into clinical practice looks promising, but validation of a universal assay with high precision for anti-PLA2R detection and defining cut-off levels are needed. Furthermore, these preliminary data should be strengthened by additional, larger studies with an adequate follow-up period.

## Figures and Tables

**Table 1:** Studies investigating the anti-PLA2R antibodies in primary membranous nephropathy.

Author	Number of patients	Method	Results
Beck et al. [10] (2009)	26	WB	Anti-PLA2R ABs: (i) Positive when there was clinically significant disease activity in 70% of primary MN cases but in none of secondary MN cases (100% specificity) (ii) Declined or disappeared in remission

Hofstra et al. [39] (2011)	18	WB	(i) 78% of MN patients were PLA2R ABs positive (ii) PLA2R ABs levels correlated strongly with both clinical status and proteinuria

Beck et al. [40] (2011)	35	WB	(i) 71% of MN patients were PLA2R AB positive (ii) Complete and partial remissions were more frequent in patients on rituximab who demonstrated decline or disappearance of PLA2R ABs (59 and 88% versus 0 and 33%) (iii) Rituximab-induced changes in antibody levels preceded changes in proteinuria

Hoxha et al. [41] (2011)	360 (100 primary MN; 17 secondary MN; 90 other GN; 153 controls)	IFA	(i) Positive PLA2R ABs had 52% sensitivity and 100% specificity to detect MN (ii) PLA2R ABs levels may help the therapeutic decisions

Hofstra et al. [38] (2012)	117	IFA and ELISA	(i) 72%/74% of MN patients were anti-PLA2R ABs positive (ii) Excellent concordance between IFA and ELISA (iii) Correlation between baseline PLA2R ABs and proteinuria (iv) Spontaneous remissions more frequent in patients with lower PLA2R AB titer (38% versus 4%)

Svobodova et al. [44] (2013)	84	IFA	Only 22% of patients in remission were PLA2R ABs positive while PLA2R AG was found in 59% of the corresponding biopsies

Kanigicherla et al. [42] (2013)	40/30 (90)	ELISA	High PLA2R ABs levels (170.8 mcg/mL) are linked with the following: (i) Active disease (ii) Higher risk of decline in renal function

Oh et al. [43] (2013)	100/69	WB	(i) 80% of nephrotic primary MN patients and 20% of patients with secondary MN were PLA2R ABs positive (ii) PLA2R ABs titer at biopsy was related to MN activity but not to outcome

Bech Anneke et al. [45] (2014)	48	ELISA	(i) PLA2R ABs decreased after two months under immunosuppressive therapy (428 U/mL to 24 U/mL) (ii) PLA2R ABs levels after 2 months of immunosuppressive therapy *not* at baseline predicted long-term outcome: 58% and 9% of negative and positive PLA2R ABs were in remission after 5 years

Hoxha et al. [46] (2014)	133	IFA and ELISA	(i) 82% of MN patients were PLA2R ABs positive (ii) Patients experiencing remission had lower PLA2R ABs titers (23 versus 54 mcg/mL) and IgG4 at baseline (iii) PLA2R ABs declined faster than proteinuria (81% versus 39% after 3 months) (iv) PLA2R ABs titer at baseline was an independent risk factor for not achieving remission of proteinuria

Segarra-Medrano et al. [47] (2014)	36		(i) PLA2R ABs titer was significantly greater in patients with remission and it preceded the clinical response (ii) No association was observed between the antibody titer prior to treatment and the mean response time or the response at 12 months (iii) Reduction in PLA2R ABs titre is significantly associated with the time until signs of remission
